# Cortical excitability and brain function in patients with insomnia: a pilot transcranial magnetic stimulation and functional near-infrared spectroscopy study

**DOI:** 10.3389/fnins.2025.1736601

**Published:** 2026-01-22

**Authors:** Jiali Luo, Qi Chen

**Affiliations:** 1 Department of Neurology, First People’s Hospital of Foshan, Foshan, China; 2 School of Psychology, South China Normal University, Guangzhou, China; 3 School of Psychology, Shenzhen University, Shenzhen, China

**Keywords:** chronic insomnia disorder, cortical excitability, functional connectivity, functional near-infrared spectroscopy, oxy-hemoglobin, short-term insomnia disorder, transcranial magnetic stimulation

## Abstract

**Background:**

Insomnia significantly impairs well-being, cognitive function, and social functioning, yet subjective psychological assessments often yield equivocal results regarding the extent of this impairment. Neural function may underlie this discrepancy and offer a more precise foundation for guiding treatment. This study therefore employed non-invasive transcranial magnetic stimulation (TMS) and functional near-infrared spectroscopy (fNIRS) to investigate cortical excitability and brain activity patterns in patients with short-term insomnia disorder (SID) and chronic insomnia disorder (CID), aiming to identify associated neural function changes.

**Methods:**

We recruited 30 patients with SID and 30 with CID. For all participants, cortical excitability was assessed by measuring the resting motor threshold (RMT) via single-pulse TMS. fNIRS was utilized to measure the concentrations of oxy-hemoglobin (Oxy-Hb) and functional connectivity in the cerebral cortex during a verbal fluency task (VFT).

**Results:**

Our study revealed that patients with SID had significantly lower RMT and higher cortical activation in the hemodynamic responses of Oxy-Hb in the bilateral dorsolateral prefrontal cortex (DLPFC), the left medial prefrontal cortex (mPFC), and the right temporal lobe (TL) than CID patients during the 60 s task period. The CID group showed significantly lower average inter-channel connectivity strength compared to the SID group. Moreover, the CID group exhibited significantly lower connectivity from the right DLPFC to the left DLPFC and the left mPFC compared to the SID group. For the SID patient group, we found that the RMT was negatively correlated with mean Oxy-Hb changes in the left mPFC. Conversely, functional connectivity between the left DLPFC and TL showed a positive correlation with RMT. Furthermore, diminished connectivity between the left TL and mPFC was associated with elevated cortical excitability.

**Conclusion:**

Patients with CID demonstrated lower cortical excitability, decreased brain activity, and reduced task-related functional connectivity relative to the SID group. This finding indicates distinct neurological profiles between short-term and chronic insomnia, a distinction that will be critical for tailoring effective neuromodulatory interventions.

## 1 Introduction

Insomnia Disorder is characterized by persistent symptoms, including difficulty initiating or maintaining sleep, or early morning awakening, that meet formal diagnostic criteria. These symptoms must also cause clinically significant distress and lead to impairments in social, occupational, or other important areas of functioning. The International Classification of Diseases, 11th Revision (ICD-11), categorizes the disorder into three subtypes: Chronic Insomnia Disorder (CID), Short-Term Insomnia Disorder (SID), and other unclassified hyposomnia. The persistence of symptoms for 3 months distinguishes chronic from short-term conditions (The Lancet, 2019). Insomnia is a complex and prevalent global health issue, affecting about 10–15% of adults (Buysse, 2013). Each year, about 27% of the population experiences SID, with roughly 72% of cases resolving on their own. A subset of individuals progresses to CID (LeBlanc et al., 2009). In China, population-based studies report a 15% prevalence of CID in the general population, rising to 35.9% among adults aged 60 and older (Lu et al., 2019). The disorder severely compromises quality of life and imposes a substantial societal and economic burden. Current medical interventions are primarily pharmacological therapies and Cognitive Behavioral Therapy (CBT) (Krystal et al., 2019; Winkelman, 2015). However, both drug-based treatments and CBT (Voelker, 2019; Kay-Stacey and Attarian, 2016; Morin, 2017) have documented limitations. Developing effective, targeted therapeutic strategies for specific insomnia subtypes is now more critical than ever.

Although SID and CID share similar symptoms, they are underpinned by distinct pathophysiological mechanisms and clinical profiles. SID typically stems from a state of acute hyperarousal, whereas CID is characterized by a failure to inhibit wakefulness, resulting in conditioned arousal (Vargas et al., 2020). Consequently, they should not be conceptualized as different stages of a single disorder, nor can CID be adequately explained by a unitary hyperarousal model. Elucidating their unique neurophysiological substrates is therefore critical, as it will pave the way for more individualized and effective interventions, ultimately improving patient care and long-term management.

Transcranial magnetic stimulation (TMS) provides a non-invasive method for assessing cortical excitability *in vivo* (Kobayashi and Pascual-Leone, 2003). When a single TMS pulse is applied to the primary motor cortex (M1), it elicits a motor evoked potential (MEP) in contralateral muscles, enabling functional evaluation of the corticospinal conduction (Di Lazzaro et al., 2001). The amplitude of this MEP serves as an index of cortical excitability. A key metric derived from this technique is the resting motor threshold (RMT), defined as the minimum stimulus intensity required to produce an MEP of >50 μV in at least five of ten consecutive trials. The RMT is believed to reflect the excitability of a central core of neurons within M1, influenced by neuronal membrane properties and non-NMDA receptor-mediated glutamatergic neurotransmission. RMT typically increases with damage to the corticospinal tract but decreases in states of cortical hyperexcitability (Rossini et al., 2015). Recently, TMS has been employed to investigate cortical excitability across various sleep disorders (Lanza et al., 2015). While existing studies consistently report altered cortical excitability compared to healthy controls, the field remains limited. The heterogeneity of sleep disorders, combined with a small number of studies, precludes a comprehensive understanding. Crucially, it remains unknown whether these changes in excitability are related to the specific neural mechanisms underlying different subtypes of sleep disorders.

Functional near-infrared spectroscopy (fNIRS) is a non-invasive optical imaging technique that measures cortical changes in oxy-hemoglobin (Oxy-Hb) and deoxy-hemoglobin (Deoxy-Hb) during brain activity, thereby detecting dynamic functional changes throughout cognitive processing (Liu et al., 2024). It offers a favorable balance between temporal and spatial resolution (Kohl et al., 2020), alongside practical advantages such as low cost and high portability. These characteristics make fNIRS particularly suitable for a wide range of experimental settings and populations, including studies conducted in dynamic or naturalistic environments (Pinti et al., 2020). Consequently, fNIRS is utilized increasingly in the auxiliary diagnosis and clinical evaluation of neuropsychological disorders. The Verbal Fluency Task (VFT) is a well-established paradigm for assessing cognitive function in individuals with neurological and psychiatric disorders (Takahashi et al., 2011). It is frequently employed in conjunction with fNIRS to monitor functional cognitive deficits (Zhang et al., 2023). The VFT engages multiple cognitive domains, including memory, language, attention, and executive function (Itakura et al., 2017). The neural correlates of the VFT involve a network of prefrontal regions, encompassing Broca’s area, the bilateral dorsolateral prefrontal cortex, and other prefrontal areas (Da et al., 2024).

This study aims to elucidate the neural profiles underlying distinct subtypes of insomnia. We hypothesize that compared to patients with SID, those with CID display reduced cortical excitability and brain function. This neural pattern is proposed to associate with a decline in prefrontal inhibitory control, which in turn contributes to their symptom of difficulty inhibiting wakefulness. To test this hypothesis, we will employ TMS to assess cortical excitability and use fNIRS to characterize brain activation and functional connectivity during a VFT. By analyzing the relationships between these neural alterations, our findings seek to provide a foundation for targeted neuromodulatory interventions for insomnia.

## 2 Materials and methods

### 2.1 Participants

**Figure 1:**
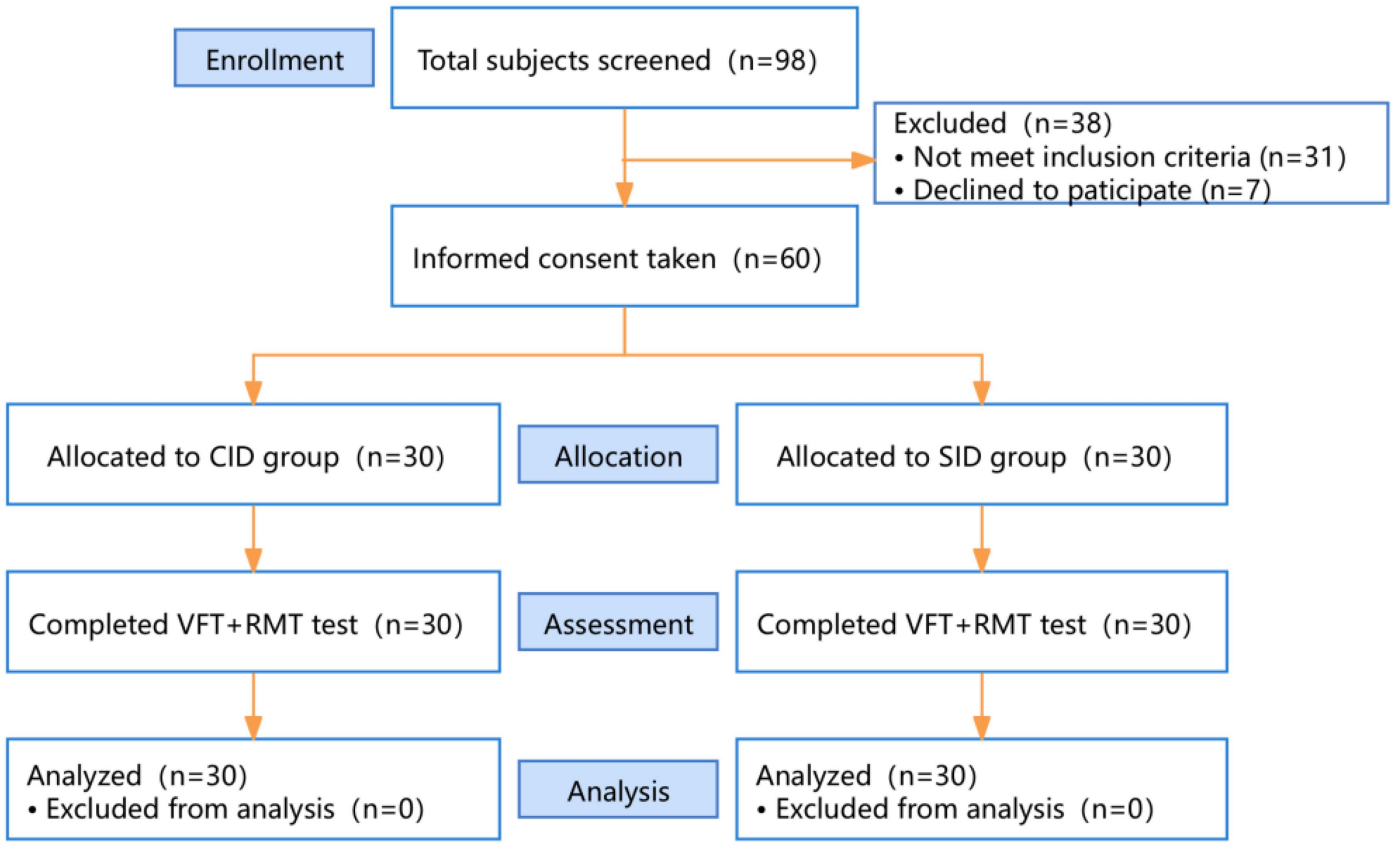
CONSORT flowchart.

This study was approved by the Ethical Review Committee of the First People’s Hospital of Foshan (FSYYY-EC-2025-96) and conducted in accordance with the ethical standards of the Helsinki Declaration. Of the 98 patients who completed screening, 31 were excluded for not meeting the inclusion criteria, and seven declined to participate. We enrolled 30 patients with SID and 30 gender-, age-, and education-matched patients with CID from the Department of Neurology, all of whom provided written informed consent. Both groups completed the experiment, and data from all 60 patients were included in the final statistical analysis (Figure 1).

The required sample size was determined using G*Power software (version 3.1.9.7). Based on the mean and standard deviation of Oxy-Hb from a pilot study (Liu et al., 2025), with a significance level (*α*) of 0.05 and a statistical power (1−*β*) of 0.8, the calculated effect size (d) was 0.749. It indicated a requirement of 29 participants per group. To ensure robust statistical validity, we recruited 30 subjects in each group.

The inclusion criteria for participants with insomnia were as follows: (1) a diagnosis according to the International Classification of Sleep Disorders, third edition (ICSD-3; American Academy of Sleep Medicine, 2014), with symptoms occurring at least 3 days per week, and a duration of either short-term insomnia (1 week to 3 months) or chronic insomnia (at least 3 months); (2) a score more than seven on the Pittsburgh Sleep Quality Index (PSQI; Buysse et al., 1989); (3) age between 18 and 68 years; (4) right-handedness; and (5) no changes to insomnia medication in the 4 weeks preceding the assessment. The exclusion criteria included: (1) a diagnosis of any serious physical illness; (2) a comorbid psychiatric or neurological disorder; (3) a history of neuromodulation therapy; (4) a history of alcohol abuse or dependence; and (5) the presence of other sleep disorders, such as obstructive sleep apnea, restless legs syndrome, or narcolepsy.

### 2.2 Verbal fluency task

The VFT protocol comprised three phases: a 30 s pre-task baseline, a 60 s task period, and a 70 s post-task baseline. During both baseline periods, participants repeatedly counted from 1 to 5. In the task phase, they were required to construct as many phrases as possible using three randomly presented Chinese characters (e.g., “大” [big], “花” [flower], “家” [home]), with 20 s allotted per character. Before the test, participants received detailed instructions to ensure smooth completion. Throughout the entire procedure, they were asked to remain seated upright and to minimize movement. The total number of correct words generated quantified their performance on the VFT.

### 2.3 Resting motor threshold

The RMT was assessed in all participants, with testing commencing 10 min after the completion of the fNIRS examination. Participants were seated in a comfortable chair with neck support and instructed to relax their right arm fully on a pillow. Electromyographic (EMG) activity was recorded from the abductor pollicis brevis (APB) muscle using Ag/AgCl surface electrodes. Single-pulse TMS was delivered using a NeuroMS/D stimulator (Neurosoft Llc, Ivanovo, Russia) equipped with a 70-mm figure-of-eight coil. The coil was held tangentially to the scalp, oriented posterolaterally at a 45° angle to the sagittal plane, with its center positioned over the left M1 to optimize the electric field strength perpendicular to the target area. The RMT was determined as the minimum stimulation intensity required to elicit MEPs of at least 50 μV in five out of ten consecutive trials (Schutter and van Honk, 2006). All procedures were conducted by a single, highly trained TMS operator with 7 years of experience, to minimize inter-operator variability.

### 2.4 NIRS measurement

**Figure 2:**
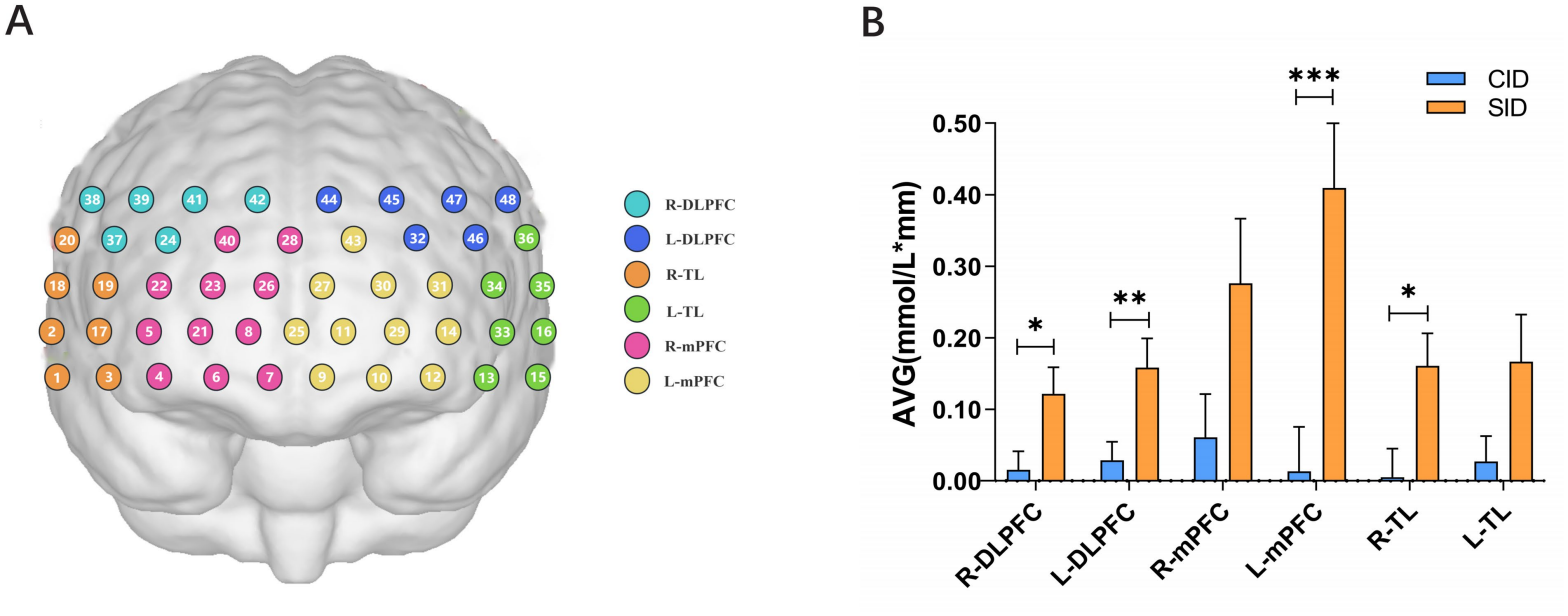
**(A)** Channels position and brain subdivisions. **(B)** Hemodynamic responses in various brain regions during the execution of the VFT. DLPFC, dorsolateral prefrontal cortex; mPFC, medial prefrontal cortex; TL, temporal lobe; R, right; L, left; CID, chronic insomnia disorder; SID, short-term insomnia disorder (*p < 0.05, **p < 0.01, ***p < 0.001, FDR-corrected).

The relative concentration changes of oxy-Hb, deoxy-Hb, and total-Hb during the VFT were measured using a 48-channel NIRS system (NirSmart-6000A, Danyang Huichuang Medical Equipment Co., Ltd., China), which applied the modified Beer–Lambert law. The system consists of 15 light source probes (emitting at 730 nm and 850 nm wavelengths) and 16 detector probes, arranged on the bilateral frontal and temporal cortical regions with an inter-probe distance of 3.0 cm. Data were acquired at a sampling rate of 11 Hz. Probe placement over the brain regions of interest (ROIs) followed the International 10–20 system for electroencephalogram electrode placement. This configuration targeted the bilateral dorsolateral prefrontal cortex (DLPFC), medial prefrontal cortex (mPFC), and temporal lobe (TL) as ROIs (Figure 2).

### 2.5 Data processing and analysis

The NirSpark software package (HuiChuang, China) was used for preprocessing and analysis of fNIRS data. Motion artifacts, which typically manifest as sudden signal jumps due to displacement between the scalp and probe, were corrected using a cubic spline interpolation algorithm. This method, based on moving standard deviation for detection, is a standard approach for mitigating such localized signal distortions (Sutoko et al., 2020). A band-pass filter (0.01–0.20 Hz) was applied to remove physiological noise, including respiration, cardiac activity, and low-frequency signal drift, thereby ensuring data stability and accuracy. Following conversion of optical densities to Oxy-Hb and Deoxy-Hb concentration changes via the modified Beer–Lambert law, subsequent analysis was restricted to Oxy-Hb based on its established higher sensitivity and signal-to-noise ratio (Wu et al., 2023).

The VFT block waveform was defined with a block range of 0–125 s, using pre- and post-baseline periods of 0–10 s and 70–125 s, respectively. The 60 s phrase construction period was used as the time window to analyze mean Oxy-Hb changes. Superimposed waveforms of Oxy-Hb changes for all patients in both groups were obtained based on individual waveforms across all 48 channels. Finally, the mean Oxy-Hb changes were averaged across the channels within each ROI.

To quantify task-based connectivity, we analyzed functional connectivity (FC) within the 60 s phrase-construction epoch. The strength of connectivity between brain regions (assessed as channel pairs) was determined by computing Pearson correlation coefficients (r) on their hemodynamic time-series. The resulting r-values were then Fisher’s z-transformed for normalization (Li et al., 2023).

Statistical analyses were performed using SPSS 27. Graphics were generated with NirSpark, GraphPad Prism 9, and Adobe Illustrator. Data normality was assessed via the Shapiro–Wilk test. Demographic group differences were analyzed using chi-square tests for categorical variables and unpaired *t*-tests or Mann–Whitney *U* tests for continuous variables. Differences in Oxy-Hb concentration across brain regions were determined with unpaired *t*-tests. FC was measured by calculating Pearson correlations between time series of channel pairs, followed by Fisher’s z-transformation. Pearson’s correlations also examined associations between Oxy-Hb changes or FC values from different brain regions and RMT. In the ROI analyses, false discovery rate (FDR) correction was applied separately to two distinct families of tests: across all ROIs for the main group contrast of task-evoked activation, and across all channel pairs within the pre-defined ROIs for functional connectivity analyses. All tests were two-tailed, with significance set at *p* < 0.05.

## 3 Results

### 3.1 Demographic characteristics

Table 1 presents the demographic characteristics of subjects in each group. Patients with CID and SID showed no difference in gender, age, education, medication, VFT performance, or PSQI scores (all *p* > 0.05). Despite this comparability, the CID group reported a significantly longer history of insomnia and exhibited a higher RMT than those with SID (both *p* < 0.05). In contrast, MEP latency and amplitude did not differ significantly between the groups (both *p* > 0.05).

**Table 1:** Demographic information of the insomnia patients.

Subjects	Mean ± SD		t/χ^2^/Z	*p* value
Variables	Chronic insomnia	Short-term insomnia		
Sample size	30	30	/	/
Gender (male/female)	12/18	17/13	1.068^b^	0.301
Age (years)	49.67 ± 12.51	44.60 ± 11.26	1.649^a^	0.105
Education (years)	10.23 ± 3.04	11.13 ± 4.18	1.251^c^	0.211
Duration (months)	48.30 ± 42.68	1.78 ± 1.02	−6.687^c^	**<0.001**
Zopiclone (no/yes)	25/5 (7.5 mg/day)	25/5 (7.5 mg/day)	0.000^b^	1.000
VFT performance	7.96 ± 1.33	8.07 ± 1.52	1.368^a^	0.129
PSQI (scores)	14.73 ± 2.50	13.90 ± 2.94	1.182^a^	0.242
RMT (%MSO)	45.13 ± 7.62	41.13 ± 5.34	2.355^a^	**0.022**
MEP latency (ms)	21.07 ± 1.18	21.73 ± 1.81	−1.666^a^	0.101
MEP amplitude (μV)	85.07 ± 25.56	87.41 ± 25.36	−0.356^a^	0.723

VFT, Verbal Fluency Task; PSQI, Pittsburgh sleep quality index; RMT, resting motor threshold; MEP, motor-evoked potential.

a Two-sample *t*-test.

b Chi-squared test.

c Mann–Whitney U test.

Significant results are highlighted (*p* < 0.05) in bold.

### 3.2 Hemodynamic responses in various brain regions

fNIRS measurements revealed significant group differences in Oxy-Hb signaling across specific brain regions (Figure 2B). Significant reductions in average Oxy-Hb were found in the CID group relative to the SID group in the following areas: the right DLPFC (*t* = −2.369, *p* = 0.021, FDR-corrected, effect size = 0.47), the left DLPFC (*t* = −2.693, *p* = 0.009, FDR-corrected, effect size = 0.59), the left mPFC (*t* = −3.577, *p* < 0.001, FDR-corrected, effect size = 0.73), and the right TL (*t* = −2.562, *p* = 0.013, FDR-corrected, effect size = 0.64). In contrast, no significant differences were found in the right mPFC (*t* = −1.987, *p* = 0.052, FDR-corrected, effect size = 0.42) or left TL (*t* = −1.863, *p* = 0.067, FDR-corrected, effect size = 0.29).

### 3.3 Functional connectivity

**Figure 3:**
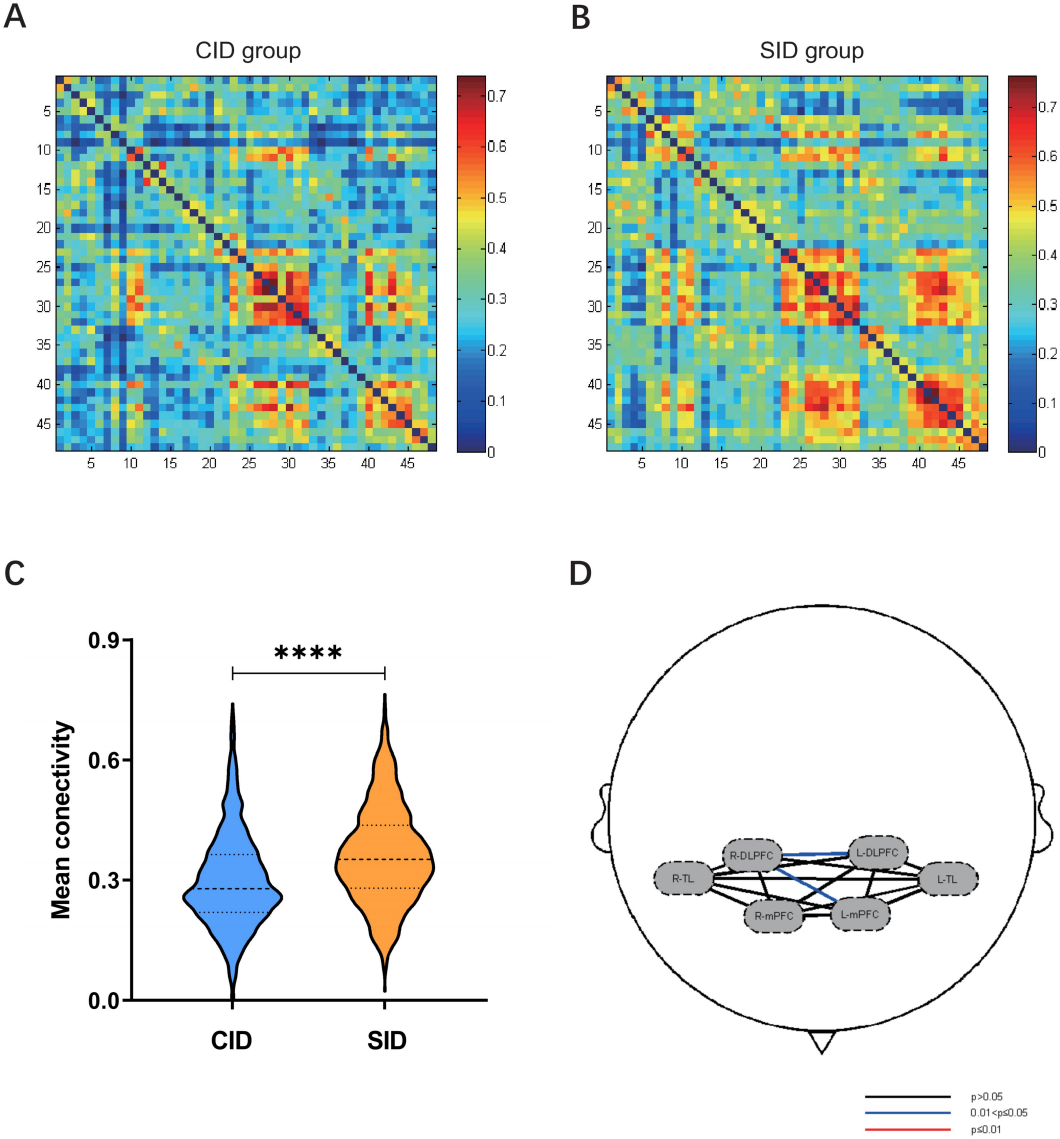
**(A,B)** The average functional connectivity strength of 48 channels in the CID group and the SID group, respectively. **(C)** The CID group had a lower mean channel-to-channel connectivity strength than the SID group. **(D)** The CID group exhibited significantly lower connectivity from the R-DLPFC to the L-DLPFC and the L-mPFC compared to the SID group (*p* < 0.05, FDR-corrected). CID, chronic insomnia disorder; SID, short-term insomnia disorder; DLPFC, dorsolateral prefrontal cortex; mPFC, medial prefrontal cortex; TL, temporal lobe; R, right; L, left; **** *p* < 0.0001.

We analyzed the strength of functional connectivity (FC) between channels in the CID and SID groups, generating 48 × 48 correlation matrices for each (Figures 3A,B). The CID group exhibited a significantly lower mean global connectivity strength compared to the SID group (*t* = −12.998, *p* < 0.0001, effect size = 0.43; Figure 3C). Subsequent regional FC analysis further identified specific pathway deficits, showing significantly reduced connectivity from the right DLPFC to both the left DLPFC and the left mPFC in the CID group (*p* < 0.05, FDR-corrected; Figure 3D). Detailed information on all different pairs of brain regions is provided in Table 2.

**Table 2:** Comparisons of connectivity between brain regions.

Brain regions	Connectivity strength		*t*	*P* value (FDR-corrected)
	Chronic insomnia	Short-term insomnia		
1–2	0.32 ± 0.21	0.44 ± 0.23	2.154	**0.035**
1–3	0.24 ± 0.17	0.33 ± 0.26	1.618	0.112
1–4	0.22 ± 0.18	0.30 ± 0.23	1.467	0.148
1–5	0.29 ± 0.19	0.39 ± 0.20	1.903	0.062
1–6	0.27 ± 0.19	0.40 ± 0.21	2.542	**0.014**
2–3	0.24 ± 0.17	0.33 ± 0.27	1.5	0.14
2–4	0.26 ± 0.19	0.29 ± 0.27	0.553	0.583
2–5	0.31 ± 0.18	0.36 ± 0.21	0.976	0.317
2–6	0.35 ± 0.19	0.43 ± 0.22	1.496	0.14
3–4	0.27 ± 0.20	0.33 ± 0.26	1.01	0.317
3–5	0.27 ± 0.16	0.34 ± 0.21	1.322	0.192
3–6	0.25 ± 0.19	0.31 ± 0.23	1.071	0.289
4–5	0.26 ± 0.17	0.30 ± 0.19	1.009	0.317
4–6	0.26 ± 0.17	0.29 ± 0.22	0.685	0.496
5–6	0.35 ± 0.16	0.38 ± 0.16	0.821	0.414

1: Right dorsolateral prefrontal cortex (R-DLPFC), 2: left dorsolateral prefrontal cortex (L-DLPFC), 3: right temporal lobe (R-TL), 4: left temporal lobe (L-TL), 5: right medial prefrontal cortex (R-mPFC), 6: left medial prefrontal cortex (L-mPFC). The data are presented as mean ± standard deviations. Significant results are highlighted (*p* < 0.05) in bold.

### 3.4 Correlations with clinical characteristics

**Figure 4:**
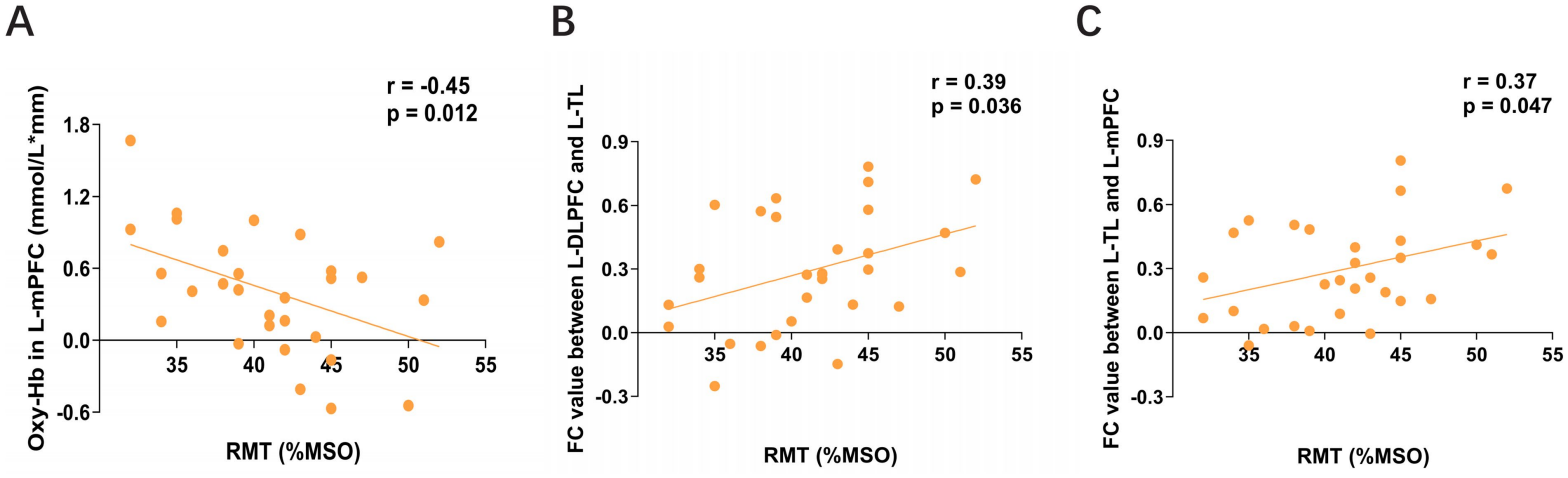
**(A)** Association between Oxy-Hb changes in the L-mPFC and RMT in the SID group. **(B,C)** Functional connectivity between different brain regions showed positive correlations with RMT in the SID group. DLPFC, dorsolateral prefrontal cortex; mPFC, medial prefrontal cortex; TL, temporal lobe; L, left; RMT, resting motor threshold; MSO, maximum stimulator output; SID, short-term insomnia disorder; FC, functional connectivity. A lower RMT reflects elevated cortical excitability in the motor cortex.

In the CID group, RMT was not correlated with hemodynamic responses in any ROI. In contrast, the SID group exhibited several significant correlations (Figure 4). RMT was negatively correlated with mean Oxy-Hb changes in the left mPFC (*r* = −0.45, *p* = 0.012). Conversely, it was positively correlated with functional connectivity between the left DLPFC and TL (*r* = 0.39, *p* = 0.036), as well as between the left TL and mPFC (*r* = 0.37, *p* = 0.047). Collectively, these findings suggest that in SID patients, diminished connectivity within a left-hemisphere frontotemporal network is associated with elevated cortical excitability.

## 4 Discussion

This study employed TMS to assess cortical excitability and fNIRS to measure hemodynamic responses during the VFT in patients with SID and CID, exploring the association between these neural metrics. To the best of our knowledge, it represents the first multimodal investigation to combine fNIRS signals with TMS-derived electrophysiological biomarkers in a population with insomnia disorder.

Research has consistently posited insomnia as a condition of central nervous system hyperarousal (Riemann et al., 2010). Evidence for this model includes PET studies demonstrating elevated whole-brain metabolism (Nofzinger et al., 2004) and event-related potential (ERP) research indicating heightened cortical arousal in individuals with insomnia during both wakefulness and sleep (Bastien et al., 2008; Yang and Lo, 2007). These findings suggest that insomnia may be characterized by altered cortical excitability. Subsequent investigations have directly probed this hypothesis. For instance, Huang et al. (2012) examined median nerve somatosensory evoked potentials (SEPs) in patients with primary insomnia (PI) and observed increased cortical excitability compared to healthy controls. In another study using TMS, van der Werf et al. (2010) found that while patients with CID exhibited a similar RMT to controls, they showed greater MEP recruitment. Building upon this work, our study reveals that patients with SID exhibit a significantly lower RMT in the left hemisphere than those with CID, indicating that cortical excitability is higher in SID.

The excitability of cortical neurons is governed by the balance between GABA-mediated inhibition and glutamate-mediated excitation (Isaacson and Scanziani, 2011). In the context of PI, previous research suggests that heightened excitability within the parietal cortex may be driven by a reduction in GABA coupled with a compensatory increase in glutamate (Winkelman et al., 2008). This model of neurotransmitter imbalance is further supported by studies of sleep deprivation in healthy individuals, which demonstrate a shift toward cortical hyperexcitability—an “activating” pattern believed to reflect changes in glutamatergic synaptic function (Kreuzer et al., 2011; Placidi et al., 2013). Given that the RMT serves as a global measure of corticospinal excitability and is dependent on glutamatergic activity (Paulus et al., 2008), it provides a valuable proxy for this neurochemical state. We posit that an imbalance in the ratio of excitatory to inhibitory neurotransmitters relates to insomnia. Furthermore, this imbalance may differ between its short-term and chronic manifestations. We emphasize that these interpretations are theoretical models proposed for future validation.

Our findings reveal that patients with CID exhibited significantly reduced Oxy-Hb concentrations in the bilateral DLPFC, left mPFC, and right TL during the VFT compared to those with SID. This aligns with a growing body of neuroimaging evidence implicating prefrontal dysfunction in CID patients. For instance, a recent fNIRS study by Zhou et al. (2024) also reported diminished prefrontal activation in CID patients relative to HCs. Further corroborating this, resting-state fMRI research by Dai et al. (2014) identified lower brain activity in the bilateral frontal lobes of insomnia patients. Structural deficits have also been observed; Joo et al. (2013) found significantly smaller grey matter volumes in multiple regions, including the bilateral DLPFC and medial frontal gyrus, in CID patients compared to HCs.

The prefrontal cortex (PFC) is a central brain structure responsible for integrating internal and external environmental information and is critical for maintaining the brain’s resting-state network. A key PFC subregion, the DLPFC, is particularly vital for higher-order cognitive processes, including emotional regulation, working memory, executive function, and language (Asgharian Asl and Vaghef, 2022). The functional integrity of the PFC is closely linked to sleep; for instance, an fMRI study by Altena et al. (2010) demonstrated reduced activation in the medial and inferior PFC of patients with CID during a VFT, providing objective evidence of PFC dysregulation in insomnia. Consequently, targeting PFC excitability has emerged as a promising therapeutic avenue. Clinical studies have shown that neuromodulation techniques like high-frequency repetitive transcranial magnetic stimulation (rTMS) and anodal transcranial direct current stimulation (tDCS), which enhance PFC excitability, can improve sleep outcomes (Zhou et al., 2020; Nardone et al., 2013). Therefore, normalizing PFC function is a significant therapeutic objective for insomnia patients, and assessing PFC activity may serve as a valuable biomarker for evaluating treatment efficacy.

This study observed no significant difference in the left TL activation between the two groups. This finding aligns with recent fNIRS research, which also reported no significant difference in brain activation or functional connectivity changes in the TL of patients with CID during a VFT compared to HCs (Zhou et al., 2024). One plausible interpretation is that patients with CID employ compensatory neural strategies; they may heighten left TL activity to counterbalance cognitive deficits, thereby sustaining normal levels of behavioral performance.

The VFT is a recognized measure of executive function, sensitive to impairments in the frontal and temporal cortices and linked to language processing (He et al., 2018; Xiang et al., 2021). Specifically, in the left hemisphere, this frontotemporal area includes Broca’s area, a region critical for language production (Amunts and Zilles, 2012). Building on this, recent research indicates that individuals with Major Depressive Disorder (MDD) exhibit reduced activation in the left frontal lobes alongside increased activation in the right frontal lobes compared to HCs (Yang J. et al., 2024). This observed hypoactivity in the left frontal lobe during VFT performance suggests a decline in its functional capacity. As a result, the heightened activity in the right hemisphere may represent a compensatory mechanism to counterbalance left-hemisphere deficiencies (Balconi et al., 2017). Interestingly, our findings indicate that during the task, the left mPFC was most activated in patients with SID, whereas the right mPFC was most activated in those with CID. While these hemispheric activation patterns in the frontal lobes align with established research about MDD, the underlying lateralization mechanisms specific to insomnia pathology require further elucidation.

Hemispheric asymmetry is considered a fundamental neural characteristic that reduces redundancy and enhances processing efficiency. This specialization facilitates parallel and flexible information processing, enabling organisms to adapt to complex neurocognitive demands (Hartwigsen et al., 2021). A classic manifestation of this division is left-hemisphere dominance for language and logical reasoning, in contrast to right-hemisphere dominance for visuospatial and emotional processing (Demaree et al., 2005; Hagoort and Indefrey, 2014; Chen et al., 2019; Goel, 2019). Converging evidence from EEG and fMRI studies indicates that depressive disorders are characterized by a disruption of this typical hemispheric activity, particularly in frontal and parietal regions. This disturbance is potentially linked to aberrant lateralization underlying cognitive and emotional functions (Grimm et al., 2008; Bruder et al., 2017).

The distinct activation profiles observed in the left mPFC and DLPFC between the two groups suggest their potential utility as neural markers for differentiating SID from CID. Consequently, interventions targeting these regions in the CID group may hold promise for alleviating insomnia symptoms and enhancing cognitive function. These findings underscore the need for more in-depth investigation into the clinical application of hemispheric asymmetry as a basis for diagnostic and therapeutic strategies in insomnia.

Notably, in individuals with SID, the RMT in the left M1 was negatively correlated with Oxy-Hb levels in the left mPFC during the VFT, suggesting that greater cortical excitability in the left hemisphere is linked to increased activation of the left mPFC during this cognitive task. In contrast, no such correlation was observed in the chronic CID group. Given that insomnia pathophysiology involves dysregulated arousal, these findings indicate that patients with SID are in a state of hyperarousal, marked by elevated cortical excitability and brain activation. Conversely, patients with CID show lower levels of both, suggesting a different physiological state. Specifically, SID appears to be triggered by hyperarousal, potentially driven by ongoing activation of the orexin system (Saper et al., 2010). The orexin system plays a fundamental role in stabilizing the sleep–wake cycle. According to the hypothalamic “Flip-Flop Switch” model proposed by Saper et al. (2005), sleep and wakefulness are two mutually exclusive, stable states that switch rapidly. This bistability arises from mutual inhibition between the wake-promoting monoaminergic systems and the sleep-promoting ventrolateral preoptic nucleus (VLPO). Orexin neurons stabilize wakefulness within this circuit by boosting monoaminergic activity, which in turn enhances cortical excitability and inhibits VLPO neurons (De Luca et al., 2022).

While CID seems to persist in association with a failure of prefrontal inhibitory control, all components of the arousal system send robust projections to the prefrontal cortex, with a particular dominance in the medial prefrontal region. From there, they project back downward to the basal forebrain, hypothalamus, and brainstem components of the arousal system (Aston-Jones and Cohen, 2005; Hurley et al., 1991). Within this circuit, reciprocal excitation rapidly enhances arousal, while mutual inhibition sustains a low-arousal state. However, a decline in the inhibitory function of the frontal cortex may also lead to an increased arousal state. Thus, chronic insomnia may be better characterized by an inability to inhibit wakefulness at sleep onset, reflecting a maladaptive dissociation between wake- and sleep-promoting systems (Buysse et al., 2011).

The proposed neural profiles may further explain the differential efficacy of pharmacotherapy between patient groups, with drugs typically being more effective for individuals with SID than for those with the CID. The most effective conventional insomnia medications, Benzodiazepine receptor agonists, function by enhancing GABAergic activity (Gottesmann, 2002). As the primary inhibitory neurotransmitter, GABA plays a fundamental role in regulating sleep–wake cycles (Siegel, 2004), and a deficit in GABAergic inhibition is strongly implicated in the hyperarousal state of insomnia (Nofzinger et al., 2004). Beyond conventional drug classes, the most recent development in insomnia treatment involves dual (DORAs) and selective (SORAs) orexin receptor antagonists. Research indicates that antagonism of the orexin receptor 2 (OX2R), which is critical for maintaining arousal, promotes sleep in a dose-dependent manner (Mogavero et al., 2023). Specifically, DORAs augment total sleep time mainly by promoting REM sleep, without affecting or even suppressing non-REM sleep (Clark et al., 2020).

Insomnia is increasingly recognized as a disorder of aberrant brain network connectivity (Nardone et al., 2020). For instance, resting-state fMRI studies using the mPFC as a seed point have identified weakened functional connectivity (FC) with the bilateral temporal lobes and left parietal lobe in insomnia patients. Dysfunctional connectivity in these regions can adversely affect the mood, memory, and cognitive function of patients with insomnia (Spiegelhalder et al., 2015). It is particularly evident in the DLPFC, where disrupted connectivity serves as a marker of cognitive decline (Vartanian et al., 2014). Recent fNIRS research has further linked specific connectivity deficits to clinical severity, showing that decreased connectivity between the left PFC and right DLPFC correlates with higher PSQI scores and poorer sleep quality in patients with SID (Wu et al., 2023). Our findings build upon this foundation by revealing a key distinction between subtypes of insomnia. We observed that patients with CID exhibited significantly lower average FC than the SID group, a difference most pronounced in pathways from the right DLPFC to the left DLPFC and left mPFC. Within the SID group, we identified a specific neurophysiological signature; diminished FC between the left DLPFC and TL, as well as between the left TL and mPFC, was correlated with elevated cortical excitability (as measured by RMT). Collectively, these results delineate distinct neural profiles for different insomnia subtypes. The SID is underpinned by reduced integrity of a left-hemisphere frontotemporal network, which is associated with heightened cortical excitability. In contrast, the progression to CID is characterized by a broader decline in functional connectivity, particularly within the bilateral prefrontal cortex.

Intra- and inter-hemispheric connectivity are thought to subserve complementary functions. Intra-hemispheric connectivity reflects functional specialization and corpus callosum inhibition, underpinning processes such as language, reasoning, and attention. In contrast, inter-hemispheric connectivity facilitates signal transmission and information integration between the hemispheres, as observed in motor control and coarse spatial processing (Gazzaniga, 2000; Wan et al., 2022). Mediated by the corpus callosum, these two patterns correspond to specialized and integrated information processing, respectively (Hartwigsen et al., 2021). We posit that the in patients with SID, a functional discoordination within the left frontotemporal region during task performance may link to diminished intra-hemispheric connectivity, which in turn triggers compensatory activation. At this stage, the division of labor between the hemispheres, mediated by corpus callosum inhibition, remains relatively intact. In contrast, patients with CID appear to experience a loss of this specialized left-hemisphere dominance. A concomitant reduction in the inhibitory function of the corpus callosum allows for greater reliance on the right hemisphere to complete the task, resulting in the observed decrease in interhemispheric connectivity. The alterations in both intra- and inter-hemispheric connectivity observed in our study may therefore reflect a pathological reconfiguration of this balance in insomnia, a compelling premise for future research. This finding is particularly significant given that similar hemispheric alterations are observed in MDD, where they predict depressive traits (Yang Y. et al., 2024). Therefore, our work provides evidence of aberrant functional hemispheric asymmetry in insomnia, advancing the pathophysiological understanding of this disorder from a novel, systems-level perspective of hemispheric lateralization.

Investigating the neural mechanisms of distinct insomnia subtypes enables a more precise, pathophysiology-driven approach to treatment. Unlike traditional pharmacotherapy, which broadly inhibits neural excitability, TMS offers the capacity for direct and bidirectional modulation of both cortical excitability and functional connectivity. Current TMS protocols for insomnia typically involve low-frequency stimulation of the right DLPFC to reduce hyperexcitability (Herrero Babiloni et al., 2021). However, our findings suggest that for patients with CID, a combined protocol incorporating high-frequency stimulation to the left DLPFC may yield superior outcomes by restoring prefrontal functionality. This hypothesis warrants rigorous validation in future clinical trials.

## 5 Limitations

This study is subject to several limitations. Firstly, the absence of a healthy control group hinders a definitive attribution of the observed neural changes specifically to the duration of insomnia and prevents the establishment of an absolute pathological deviation from a normative baseline. Secondly, future studies should incorporate neuronavigated, bilateral RMT measurements to investigate potential interhemispheric excitability differences with greater precision. Thirdly, the spatial coverage of our fNIRS system was limited; future investigations should target whole brain regions, especially the motor cortex. Fourthly, the reliance on subjective questionnaires for participant selection introduces potential bias, as it excludes objective measures like polysomnography. Finally, given the established interplay between insomnia and emotional states, incorporating standardized anxiety and depression assessments in future research would enable a more comprehensive evaluation.

## 6 Conclusion

Our results reveal that patients with CID demonstrate lower cortical excitability, decreased brain activity, and reduced task-related functional connectivity compared to the SID group. These findings suggest that suppressed blood oxygen metabolism in the cerebral cortex may be related to the pathogenesis of CID. Notably, in SID patients, higher cortical excitability was significantly correlated with greater activation of the left mPFC. Furthermore, cortical excitability in SID patients was significantly correlated with the degree of disrupted connectivity strength between the left frontal and temporal lobes. Consequently, future research should employ fNIRS and TMS to support clinical screening and optimize therapeutic interventions for insomnia.

## Data Availability

The raw data supporting the conclusions of this article will be made available by the authors, without undue reservation.
